# Illinois State Medical Society

**Published:** 1851-07

**Authors:** A. L. M’Arthur

**Affiliations:** Secretary Ill. State Med. Society


### Part 4.—Editorial and Miscellaneous

### ARTICLE I.

### ILLINOIS STATE MEDICAL SOCIETY.

The Illinois State Medical Society met at the City of Peoria, in the spacious court house, at 11 o'clock A. M. on the 3d of June, 1851, the President, Dr. Wm. B. HERRICK, of Chicago, in the Chair. The Constitution and By-Laws were read by the Secretary.

The Committee of Arraugements reported twenty-one delegates from Auxiliary Societies and Medical Institutions within the State.

On motion of Dr. Joseph C. Frye, of Peoria, the delegates from the several counties represented were directed to select one of their number to act as a committee to nominate officers for the ensuing year.

The following gentlemen were elected that committee:

Drs. J. W. Spalding, of Stark County Medical Society; S. A. Paddock, of Upper Illinois Medical Society; H. Shoemaker, of Monroe Medical Society; E. McArthur, of Chicago Medical Society; S. Thompson, of Esculapean Medical Society; L. G. Thompson, of Upper Illinois Medical Society; A. L. McArthur, of Will County Medical Society; J. C. Fitz, of Fulton County Medical Society; C. W. Andrews, of Winnebago County Medical Society.

On motion of Dr. E. S. Cooper, of Peoria, the Society adjourned to 2 o'clock P. M.

*Afternoon Session.*

Dr. Wm. B. Herrick in the Chair, the Committee appointed for the purpose of nominating officers reported the following:

*President.*—S. THOMPSON, of Albion.

*Vice Presidents.*—E. M'ARTHUR, of Chicago, and T. HALL, of Stark.

*Secretaries.*—H. SHOEMAKER, of Monroe, and A. L. M'ARTHUR, of Joliet.

*Treasurer.*—R. ROUSE, of Peoria.

The report was adopted and the nominees unanimously elected.

The President elect was conducted to the Chair by the Chairman of the Committee on Nominations. He made a short and appropriate address.

On motion of Professor Herrick, Dr. Max Myers, of Chicago, was elected member by invitation.

A long and interesting discussion arose here in respect to the qualifications requisite for membeeship, those present expressed themselves freely upon this question, and in order to give form to their views, on motion of Dr. C. N. Andrews, the qualification of membership was referred to a Committee of five with instructions to report in the morning session.

Drs. J. C. Frye, R. Boal, Wm. B. Herrick, C. N. Andrews and S. A. Paddock were appointed that Committee.

On motion of Dr. J. C. Frye, the meeting adjourned to 8 o'clock P. M., to hear Ex-President Herrick's valedictory address.

*Evening Session.*

The Court House was filled with members and citizens who had been invited to attend. The address was listened to with much attention, and abounded in original thought and practical suggestions.

On motion the Society adjourned to 8 o'clock to-morrow morning.

*Morning Session.*

Dr. S. Thompson, President, in the chair, the Committee on the qualifications of members reported the following amendment to the constitution under article 2d. The section touching membership shall be amended so as to read as follows :

" The delegates shall receive their appointment from permanently organized Medical Societies, Medical Colleges, Hospitals, Lunatic Asylums, and other permanently organized institutions of good standing in the State of Illinois. Each delegate shall hold his appointment for one year and shall participate in all the business and affairs of the Society."

" The *permanent members* shall consist of regular graduates from reputable schools of medicine who have served in the capacity of delegates, or such other regular graduates as may receive the appointment by unanimous vote."

" Permanent members shall at all times be entitled to attend the meetings and participate in the transactions of the Society so long as they continue to conform to its regulations, and when not in attendance they shall be authorized to grant letters of introduction to reputable practitioners of medicine residing in their vicinity, who may wish to participate in the meetings as provided for members by invitation."

Laid over to the next annual meeting of the Society.

Thirty nine M. D's. were proposed as permanent members and were unanimously elected.

The reports of standing Committees being in order, Dr. E. S. Cooper, in the absence of the chairman of the Committee, on Practical Surgery, read a paper which he intended to add to the report, on the beneficial effects of chloroform as an anæsthetic agent in operative surgery. Referred to Committee on Publication.

Dr. S. Thompson, chairman of the Committee on Practical Medicine, read an elaborate report. Referred to Committee on Publication.

On motion of Dr. Chamberlain, the Society adjourned to 2 o'clock P. M.

*Afternoon Session.*

The Society proceeded to select a place for holding the next annual meeting. Jacksonville and Chicago were put in nomination, and after an interchange of opinion of the advantages of those places respectively in regard to facility of access and the best interest of the profession throughout the State. Jacksonville was finally selected.

On motion of Dr. A. L. Mc'Arthur, a Committee of three was appointed by the chair to nominate members for the Standing Committees.

Drs. E. M'Arthur, Dickinson and S. A. Paddock, were appointed that Committee.

Dr. C. N. Andrews moved the following:

Resolved, That this Society again recommend the organization of County Medical Societies throughout the State, and in case it is found inconvenient for single counties to organize several counties are recommended to join for that purpose.

Dr. Wm, B. Herrick gave notice of the following amendment to the Constitution.

Resolved, That the Constitution of this Society be so amended as to strike out all relating to the appointment and duties of Committees on Practical Medicine, Surgery, Obstetrics, and Drugs and Medicines, and that there be established a provision for the appointment of at least four special Committees, each consisting of three members, the duties of which it shall be to investigate and report upon general or special subjects such as may be selected by a vote of the Society. Lies over until the next annual meeting of the Society.

The Committee on nominations of Standing Committees reported the following:

Drs. E. Roe, I. Richardson and J. A. Halderman Committee on Arrangements.

Drs. N. S. Davis, H. Shoemaker and T. Hall Committee on Practical Medicine.

Drs. —— Murphy, Wm. B. Herrick and A. L. Mc'Arthur, Committee on Surgery.

Drs. R. Boal, J. C. Fitz and J. A. Haldeman Committee on Obstetrics.

Drs. J. V. Z. Blaney, J. C. Frye and W. E. Moss Committee on Drugs and Medicines.

The report was adopted and the gentlemen in nomination unanimously elected.

Dr. Rouse being present, in the absence of the Chairman on Obstetrics, read a paper which he has hastily prepared on the use of Chloroform in parturituon. Referred to Committee on Publication.

On motion of Dr. R. Rouse, the Society proceeded to the election of delegates to the National Medical Association which holds its next session at Richmond, Va.

The following gentlemen were elected :

Drs. R. Rouse, C. N. Andrews, J. D. Arnold, H. Shoemaker and S. Thompson ; and as alternates, Drs. E. M'Arthur, E. Dickinson, L. G. Thompson and E. S. Cooper.

On motion of Dr. R. Boal, Dr. A. E. Ames and Dr. J. C. Frye were appointed a Committee to prepare a report on the registration of births, deaths and marriages ; the Committee formerly elected having failed to report.

Dr. E. M'Arthur presented a preamble and resolutions to the effect, that the time had come when the people of the State of Illinois ought to regulate by statutory enactment, the qualifications of those who practice medicine and surgery.

Dr. E. S. Cooper offered a preamble and resolutions in reference to the present laws and public sentiment, which make the surgeon legally responsible for the performance of operative surgery; but, at the same time, are hostile to those means by which the requisite knowledged is obtained for the skillful performance of his duty. Referred to the Committee on Publication.

Dr. J. C. Frye offered the following resolution which was laid upon the table.

Resolved, That a Committee of three be appointed to memoralize the Legislature in regard to the unjust and oppressive operation of the late law of homestead exemption upon the medical profession; seeing that the mechanic is secured in his remuneration for labor necessarily completed before payment, and while the merchant possesses the choice of saying whom he will trust, the physician has to attend all, perhaps more because they are poor, and under the present law is dependent wholly upon the honor of a large number who are indebted to him and never intend to pay.

During the whole session there was manifested the best feeling between the members, in the discussions of the varied topics introduced.

After the passage of a vote of thanks to the Committee of Arrangements and the faculty of Peoria, for the kindness and attention manifested to all, the Society adjourned *sine die.*

A. L. M'ARTHUR,

*Secretary Ill. State Med. Society.*

---

ARTICLE II.

